# Evaluating a longitudinal point-of-care-ultrasound (POCUS) curriculum for pediatric residents

**DOI:** 10.1186/s12909-021-02488-z

**Published:** 2021-01-19

**Authors:** Julia Aogaichi Brant, Jonathan Orsborn, Ryan Good, Emily Greenwald, Megan Mickley, Amanda G. Toney

**Affiliations:** 1 Department of Pediatrics, Section of Emergency Medicine, University of Colorado/Children’s Hospital Colorado, 13123 E 16th Ave, B251, Aurora, CO 80045 USA; 2 Department of Pediatrics, Section of Pediatric Intensive Care, University of Colorado/Children’s Hospital Colorado, Aurora, CO USA; 3 Department of Pediatrics, Denver Health Medical Center, Denver, CO USA

**Keywords:** POCUS, Medical education, Pediatrics, Curriculum development, Emergency medicine

## Abstract

**Background:**

POCUS is a growing field in medical education, and an imaging modality ideal for children given the lack of ionizing radiation, ease of use, and good tolerability. A 2019 literature review revealed that no US pediatric residency programs integrated obligatory POCUS curricula. Our objective was to provide a formalized POCUS curriculum over multiple years, and to retrospectively assess improvement in resident skills and comfort.

**Methods:**

During intern year, pediatric residents received didactics and hands-on scanning opportunities in basic POCUS applications. Their evaluation tools included pre- and post-surveys and tests, and a final performance exam. In the second and third years of residency, all participants were required to complete 8 hours per year of POCUS content review and additional hands-on training. An optional third-year curriculum was offered to interested residents as career-focused education elective time.

**Results:**

Our curriculum introduced POCUS topics such as basic and advanced cardiac, lung, skin/soft tissues and procedural based ultrasound to all pediatric residents. Among first-year residents, application-specific results showed POCUS comfort level improved by 61–90%. Completed evaluations demonstrated improvement in their ability to recognize and interpret POCUS images. Second- and third-year residents reported educational effectiveness that was rated 3.9 on a 4-point Likert scale. Four third-year residents took part in the optional POCUS elective, and all reported a change in their practice with increased POCUS incorporation.

**Conclusions:**

Our longitudinal pediatric residency POCUS curriculum is feasible to integrate into residency training and exhibits early success.

**Supplementary Information:**

The online version contains supplementary material available at 10.1186/s12909-021-02488-z.

## Introduction

Point-of-care ultrasound (POCUS) is an ideal imaging modality for many reasons including portability, allowing for immediate bedside access, assistance in real time decision making, and procedural accuracy. Many medical specialties use ultrasound in some manner, including anesthesia [1], neonatology [2], internal medicine [3–5], family medicine [6, 7], critical care medicine [8–10], and emergency medicine [11, 12].

Despite the obvious benefits including safety improvements and lack of ionizing radiation, most pediatric residency training programs have been slow to adopt POCUS as part of their curriculum. In 2015, the American Academy of Pediatrics (AAP) endorsed the incorporation of POCUS training into pediatric emergency medicine (PEM) fellowships, as well as POCUS education for PEM physicians [13]. A 2020 survey of national PEM fellowship program directors reported 77% of PEM fellowship training programs have an ultrasound curriculum, a significant increase from 10 years ago [14]. The biggest barrier identified by the survey was lack of qualified faculty as educators. In 2019, the PEM POCUS Network, a multinational group of pediatric and emergency physicians dedicated to promoting clinical use of POCUS, defined a curriculum standard for PEM fellowships and POCUS core content for PEM POCUS fellowships [15].

Prior to these efforts, no specific consensus existed for POCUS applications in these programs. Despite the advancements in subspecialty POCUS education, in 2019 a national survey of internal medicine, combined internal medicine-pediatrics and pediatric residency programs demonstrated that there were no pediatric residency programs with a required POCUS curriculum, and < 15% of pediatric residency programs offered an optional curriculum [16].

Given the growing use of POCUS, as well as its benefits to the pediatric population and expansion into pediatric subspecialties, we developed a mandatory, longitudinal POCUS curriculum for all pediatric residents. One goal of this study was to design and actualize a three-year POCUS curriculum for that was feasible to teach to pediatric residents, and a second goal was to retrospectively evaluate its implementation. In doing so, we hope to offer other pediatric residency programs a framework of how to design their own POCUS curriculum.

## Methods

This study was conducted at two pediatric centers in Colorado—one is a Pediatric ED (PED) within a urban safety-net hospital and the other is a large, free-standing, tertiary academic children’s hospital. Residents rotate through both hospitals during their three-year training.

Prior to curriculum implementation, a needs assessment sent to pediatric residents demonstrated a strong interest in POCUS, but a lack of formal training. To address that training deficit, a basic POCUS course was introduced to first-year residents in January 2017. This curriculum was developed by a POCUS fellowship trained PEM physician and integrated into first-year residents’ PED experience [17].

The POCUS curriculum consisted of three half-days of education over a three-month period (a total of 12 contact hours). Prior to the start of the curriculum, all residents took a pre-survey and pre-test to evaluate their baseline comfort with and knowledge of POCUS. The first two half-days of education included 1 hour of lectures and video review, followed by 3 hours of hands on scanning. Topics covered were: how to use the ultrasound machine (knobology), extended focused assessment for trauma (e-FAST), soft tissue, basic cardiac, and resuscitation ultrasound. The third half-day included a post-test to evaluate learning retention, as well as an objective structured clinical exam (OSCE).

The pre-course survey collected information regarding demographics, former POCUS training, POCUS comfort level, and frequency of ultrasound use in the past 3 months. Frequency of use was defined as often (> 1 time per week), somewhat often (1–2 times per month) and occasional (< 1 times per month). Following the pre-survey and pre-test, the first educational session focused on basic knobology and e-FAST.

The goal of E-FAST session was for residents to recognize the presence or absence of intra-abdominal fluid and/or pericardial fluid, as well as identify the presence or absence of lung slide, B-lines and correctly identifying lung point.

The second session consisted of a didactic addressing soft tissue and musculoskeletal POCUS including the following content: identifying normal soft tissue and musculoskeletal structures, identifying and differentiating types of foreign bodies, and identifying an abscess. The facilitators created a home-made model from tofu to simulate foreign body and abscess identification.

The third session focused on basic cardiac and resuscitative ultrasound. The goal of this session was for residents to recognize the presence or absence of cardiac activity and a pericardial effusion, and how to identify and assess the inferior vena cava. Residents then had 3 hours for hands-on practice with these views, again using each other as subjects. At the conclusion of the session, participants completed a post-test, a post-survey, and an OSCE on the FAST to evaluate their ability to accurately acquire images. The post-course survey collected comfort level data, frequency of ultrasound use in the past 3 months and an evaluation of the curriculum.

Testing materials were created for this curriculum by the POCUS director at the safety-net hospital. The pre- and post-written test consisted of 38 questions, including video-based questions on ultrasound techniques and image interpretation. The test was modeled after the Online Emergency Ultrasound Exam produced by the Emergency Ultrasound Section of ACEP [18]. The OSCE evaluated each resident’s ability to acquire FAST images on a model. The OSCE evaluators were POCUS-trained physicians who completed an OSCE training course. A 31-item checklist was derived from the standardized direct observation tool for the FAST exam developed by the Academy of Emergency Ultrasound [19]. Only residents’ ability to perform a FAST exam was evaluated, not the e-FAST, as the tool used that was reviewed and validated by Academy of Emergency Ultrasound focused only on the FAST.

Given the resident feedback from the first-year curriculum, the curriculum was broadened and expanded to second- and third-year residents (Table 1). For the second- and third-year pediatric residents, a new component of the POCUS curriculum was incorporated into required academic half-day sessions 1–2 times per year at the free-standing, academic pediatric hospital. Residents rotated through stations with hands-on review of the e-FAST, the focused cardiac exam, ultrasound guided procedures, and basic lung ultrasound alongside POCUS-trained faculty. Topics varied based on resident suggestions, as well as relevancy to PED or pediatric intensive care unit (PICU). The curriculum was evaluated by resident feedback for each session.

**Table 1:** POCUS Curriculum Design

First-year	Second−/Third-year	Optional Third-year
**e-FAST:** **presence of intraabdominal fluid, pericardial fluid, lung slide**	**e-FAST:** **presence of intraabdominal fluid, pericardial fluid, lung slide**	**e-FAST:** **presence of intraabdominal fluid, pericardial fluid, lung slide**
**Soft tissue:** **Cellulitis, abscess identification**	**US guided procedures:** **Central lines, arterial lines, US-guided IVs**	**US guided procedures:** **Central lines, arterial lines, US-guided IVs**
**Musculoskeletal (MSK):** **Joint effusion, fractures, myositis**	**Basic cardiac:** **4 views, gross EF, presence of absence of fluid**	**Advanced cardiac:** **4 views, gross ejection fraction, presence of absence of fluid, IVC measurement, septal flattening, chamber size, regurgitation**
**Basic cardiac:** **4 views, gross EF, presence of absence of fluid**	**Lung:** **Lung slide, lung point, B-lines, pleural effusion, subpleural consolidations**	**Abdominal emergencies:** **Appendicitis, intussusception, pyloric stenosis, SBO**
**Resuscitation:** **Gross evaluation of IVC pre and post-fluid, cardiac function**		**Lung:** **Lung slide, lung point, B-lines, pleural effusion, subpleural consolidations**
		**MSK:** **Joint effusion, fractures, myositis**
		**Skin/soft tissue:** **Cellulitis, abscess identification and plan for drainage with measurements and flow**
		**Nerve block:** **Fascia iliaca nerve block, posterior tibial, forearm blocks and anterior scalene**

In 2019, third-year residents were offered an optional one-week elective in advanced ultrasound topics. These residents completed the elective in April–June 2019. They spent eight half-days learning ultrasound-guided procedures, scanning and reading with echo-trained cardiologists, scanning with ultrasound technologists, and participating in didactics and scanning with members of the PED POCUS faculty at both hospitals (Table 2). They were sent a similar post-test and course evaluation that the first-year residents received at the end of their POCUS rotation to assess the course.

**Table 2:** Sample third-year Curriculum Schedule

	Subject or Application (Location)				
	Monday	Tuesday	Wednesday	Thursday	Friday
**AM**	**POCUS review (e-FAST) and Peds Abdomen** **(PED)**	**Lung and independent scanning** **(PED)**	**ECHO/advanced cardiac** **(Cardiology)**	**Independent scanning/nerve blocks** **(PED)**	**Scanning with US tech** **(Radiology)**
**PM**	**US guided procedures and advanced cardiac evaluation** **(PICU)**	**Clinic**	**Academic half-day**	**Skin/soft tissue/MSK** **(PED)**	**Video review and wrap-up** **(PED)**

Topics for the third-year residents did vary slightly, depending on the area of interest for each resident. Residents applying for PICU fellowship spent more time in the PICU and with the cardiology specialists. Emergency medicine-bound residents focused more on skin/soft tissue, nerve blocks, and pediatric abdominal emergencies such as appendicitis, intussusception, pyloric stenosis and intestinal obstruction. All PED instructors were POCUS-fellowship trained faculty. Additionally, these third-year residents were given time to independently scan in the ED, with all scans reviewed alongside POCUS faculty members.

### Statistical analysis

Pre-test, post-test, and OSCE scores were calculated by dividing the number of questions answered correctly by the number of questions on the test (38 questions for the pre-test and post-test, 31 for the OSCE). Age, pre-test, post-test and OSCE were presented as means with standard deviations (± sd) as their distributions were found to be normal. Mean difference with 95% confidence intervals were given for two group comparisons. Relative risks with 95% confidence intervals were calculated to describe the relationship between prior training and number of prior exams performed to comfort level acquiring and interpreting exams. Prior training (yes versus no), number of exams performed (0–10 exams versus > 10 exams), and comfort level (comfortable vs uncomfortable) were dichotomized for analysis. Chi-square and Fisher’s exact tests were used to analyze categorical variables. The Student’s t-test was used to analyze continuous variables. The paired t-test was used to compare the change from pre-test to post-test. McNemar’s test was used to compare categorical variables from the pre-course survey to the post-course survey. A *p*-value of ≤0.05 was considered statistically significant. Statistical analyses were performed with SPSS version 22 (IBM SPSS, Armonk, NY, US). The study was approved, and informed consent was waived by the local institutional review board.

## Results

**Fig. 1:**
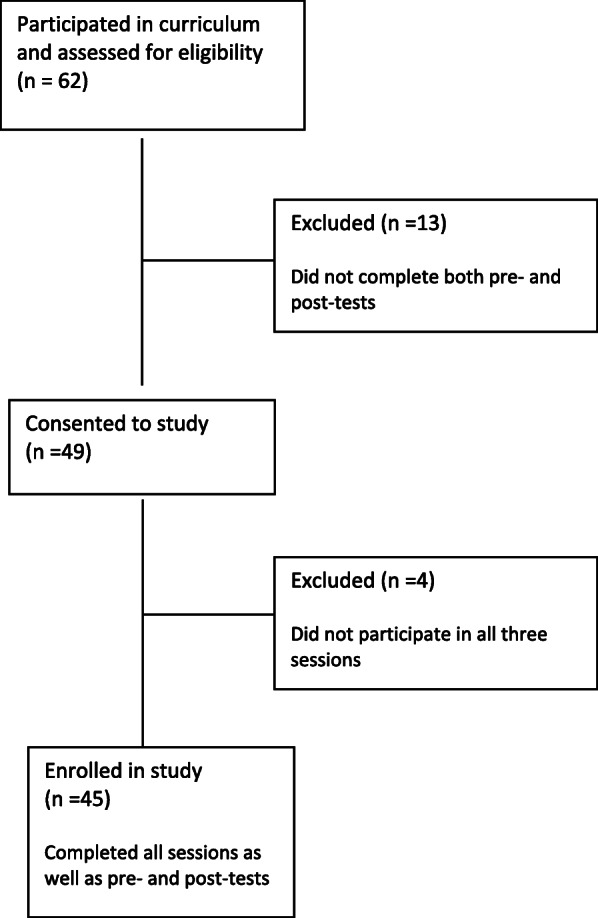
Eligibility and Enrollment for first-year residents

Ninety-five pediatric residents completed the initial needs assessment in 2016. Between January 2017 and March 2019, 62 first-year pediatric residents participated in the curriculum; however, only a total of 45 were eligible for the study (Fig. 1). None of the participants completed a prior residency training program.

**Fig. 2:**
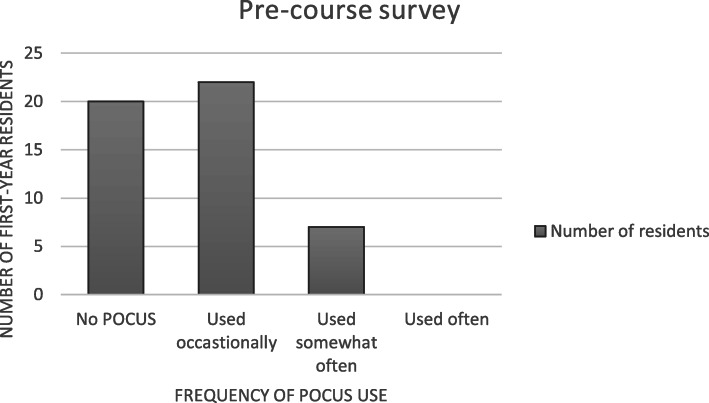
Results of pre-course survey of first-year resident POCUS use

On the pre-course survey, first-year residents self-reported that their POCUS use 1) never used POCUS in any clinical setting, 2) occasionally used it (<one time per month), 3) used it somewhat often (one to two times per month or 4) used often (> one time per week) (Fig. 2). Most first-year pediatric residents (84%) reported that they had performed 0–10 POCUS exams prior to the POCUS curriculum.

**Fig. 3:**
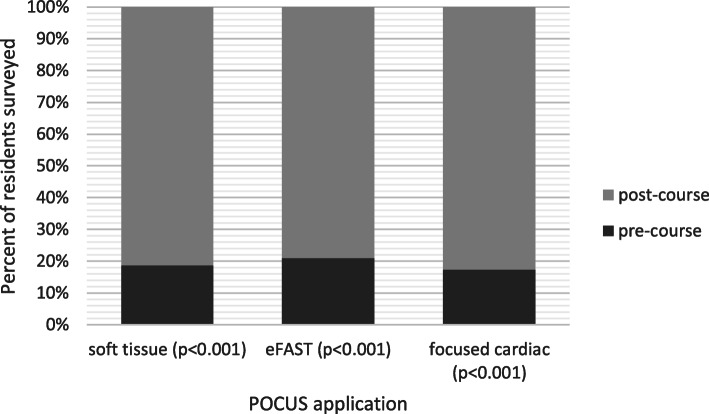
Results of first-year resident comfort level before and after POCUS education

The mean pre-test score was 68 (± 8.5) and the mean post-test score was 83 (± 8.3) with a difference of 15 (95% CI: 12.5–17.6, *p* < .001). Mean FAST OSCE performance after completion of the curriculum was 88.7 (± 11.9). First-year pediatric residents reported overall improvement in comfort level of all modalities (Fig. 3). However, the majority reported independent completion of five or fewer soft tissue exams (45/49, 92%), five or fewer e-FAST exams (46/49, 94%), and five or less cardiac exams (48/49, 98%) during the three-month educational period.

All first-year participants stated they found the curriculum useful and 42/49 (86%) stated the teaching was very effective with the remaining seven (14%) participants stating the teaching was somewhat effective. When asked to state their preference for additional training, participants preferred hands-on sessions (43, 88%), review of US images with experts (28, 57%), classroom didactics (15, 31%) and web-based teaching (10, 20%).

The academic-half day data included an evaluation of teaching effectiveness on a 4-point Likert scale (1 = not effective, 4 = very effective). Neutral was not included as part of this scale. Collectively, POCUS teaching effectiveness was rated 3.9/4 by the 63 s-year and third-year residents who participated.

The third-year residents who participated in the POCUS elective did not take part in a pre-survey but did complete a post-course survey in October 2019, 4–6 months after conclusion of the elective. Three of the four residents who participated reported feeling somewhat uncomfortable or neutral in their comfort level with ultrasound prior to the course. After the elective, all four residents reported improved comfort level in ultrasound to somewhat comfortable (75%) or very comfortable (25%). All four third-year residents reported using POCUS during their clinical practice since the elective. Additionally, all four third-year residents would recommend the course to a colleague.

Looking across the years, these four residents all participated in the initial evaluation of the first-year curriculum when it was first implemented in 2017. All four third-year residents felt that the longitudinal POCUS curriculum was useful overall, and that the education they received had or would change their how often they incorporate POCUS into their practice.

## Discussion

Our curriculum was designed to offer organized, interdisciplinary POCUS training to pediatric residents throughout all 3 years of their training recognizing the importance of ultrasound as an imaging modality in children. Few other pediatric residency programs offer a POCUS curriculum, and none are compulsory or longitudinal [16]. Other residency programs such as emergency medicine and internal medicine that have incorporated POCUS education into core curricula have demonstrated benefits from a longitudinal model, given the ability of trainees to retain and reuse core concepts [20].

Our primary aim was the development of the curriculum as a mandatory component of education for pediatric residents. Our second aim was to retrospectively review and evaluate its implementation among our residents. Another aim was to establish a frame of reference for other pediatric residency programs interested in developing or expanding a POCUS curriculum.

The first-year curriculum evaluation demonstrated that first-year pediatric residents were able to learn and retain basic POCUS after three half-day educational sessions. Didactics combined with hands-on scanning time led to a high percentage of successful scan completion and improved comfort level with POCUS. Despite this progress, no resident showed an increase in independent scans performed during the three-month elective period. We suspect one reason for this is the reality that many pediatrics attendings are not POCUS trained, and thus do not feel comfortable actively supporting POCUS use. For example, at our free-standing, academic children’s hospital, only three of 91 faculty members (PEM physicians and pediatricians) are credentialed in POCUS. As discussed previously, the most significant barrier to more frequent POCUS use is lack of qualified educators [14]. Although ACEP and the ACGME have recently recommended training for PEM fellows in structured POCUS education, the minority of current PEM attendings report having receiving formal training. As new PEM faculty enter the work force, this will likely change. A survey from 2006 reported just over 50% of PEM fellowship programs had faculty who used any POCUS in the PED [21], and prior to 2011 POCUS education available to PEM fellows was limited [22, 23]. Often pediatric trainees enter residency with more POCUS experience than their attendings, but based upon our needs assessment they feel uncomfortable using POCUS for patient care without supervision. Additionally, the first-year study showed that, per resident self-report, few POCUS exams were done outside the training sessions during the study period. As a result, we suspect these trainees will likely lose their skills and will not be able to retain their POCUS knowledge beyond the short-term. One way to improve this anticipated decline in knowledge would be to create training programs for pediatric attendings, providing residents with more opportunities to scan in the clinical environment.

Second- and third-year residents felt they received effective teaching and appreciated the hands-on didactic experiences, but no other formal pre- and post-course data was collected. Scans performed by pediatric residents are not tracked; as such, we do not have data establishing whether residents used their POCUS skills independently, and if so how long after didactics and hands-on training they maintained those skills. As a result, while we can confidently say that our education is effective in the short-term, we cannot assert that translates into long-term knowledge or increased POCUS use. A 2019 study demonstrated that residents can lose their POCUS skills in as quickly as 4 weeks without repetition, depending on the application type [24]. Further study is needed to evaluate our residents’ retention of POCUS skills throughout their 3 years of training to better evaluate the effectiveness of this longitudinal program.

The four third-year residents in the POCUS elective found the curriculum valuable and 100% reported improved POCUS comfort level. All reported utilizing POCUS clinically after residency completion, possibly because those residents entered fields where POCUS is more commonly used. Two residents are now in PEM fellowship, one is in PICU fellowship, and one is working in an urgent care setting. All locations provide the opportunity to apply POCUS.

Our curriculum was relatively easy to develop given pre-existing materials on POCUS education and access to POCUS trained faculty, and we were able to incorporate it into the residents’ required education during their ED rotation months. Overall, all 3 years of pediatrics residents found the teaching effective and valuable. The expansion of POCUS into other pediatric fields such as neonatology, sports medicine, rheumatology, hospitalist medicine, as well as its growing use in PEM, PICU and anesthesia, provide ample opportunity for pediatric residents to continue the application of clinical POCUS. To facilitate that implementation, training programs including both didactic and hands-on education must be developed for both trainees and pediatric attendings to improve their POCUS skills and comfort level.

### Limitations

Limitations of the first-year component of this study included: use of single institution data, and lack of exposure to pediatric anatomy and physiology, as only adult volunteers were scanned. The first-year residents who were evaluated did not perform a pre-training OSCE so we do not have scores for comparison, but given their inexperience with POCUS it is likely there would still be an improvement in post-training OSCE scores.

For the second-year residents’ curriculum, we did not have a formal pre-and post-evaluation and instead relied on subjective feedback in writing as well as one measure of effective teaching. To better evaluate our longitudinal curriculum, we need to establish an assessment tool similar to the first-year and third-year models, and consider developing a method to track independent POCUS-scans outside of didactics. This is especially true given concerns regarding loss of POCUS skills over time. Additionally we only used a 4-point Likert scale for evaluation of teaching during academic half-day without a neutral option, as this required our residents to commit to an answer. However, this forces residents to answer and does not offer a truly neutral option therefore potentially inflating our results.

Lastly, there was a small sample size of third-year residents, all of whom had selected career paths in fields where POCUS is utilized. Given we studied curriculum effectiveness in a population of self-motivated and interested residents, we cannot generalize our results from the elective to all pediatric residents. Therefore, selection bias must be factored in to the POCUS elective resident data.

## Conclusions

POCUS is a burgeoning imaging modality in many fields of medicine given mounting evidence of its contributions to improving safety and efficiency in clinical care. Pediatrics is no exception. With the growing incorporation of POCUS into undergraduate and graduate medical education, it would be in the best interest of pediatric residency programs to develop a standardized POCUS curriculum. Longitudinal POCUS education for pediatric residents is vital, easy to implement, well received, and residents self-report feeling more comfortable with POCUS use. Opportunities for continued POCUS learning and practice to improve resident knowledge and skill retention is needed, highlighting the fact that enhanced POCUS education for pediatric attendings is also critical.

## Supplementary Information

**Additional file 1.** Pediatric Resident Ultrasound Curriculum – Pre-Test.

**Additional file 2.** Pediatric Resident Ultrasound Curriculum – Post-Test.

**Additional file 3.** Pediatric Resident Ultrasound Curriculum - Pre-course Survey.

**Additional file 4.** Pediatric Resident Ultrasound Curriculum - Post-course Survey.

**Additional file 5.** Pediatric Resident Ultrasound Curriculum – OSCE.

## Data Availability

The datasets used and/or analyzed during the current study are available from the corresponding author on reasonable request. Supplemental materials including pre/post-tests are available with this manuscript. All methods were carried out in accordance with relevant guidelines and regulations.

## Abbreviations

POCUS: Point-of-care ultrasound
ACEP: American College of Emergency Physicians
AAP: American Academy of Pediatrics
PEM: Pediatric emergency medicine
PED: Pediatric ED
e-FAST: Extended focused assessment for trauma
OSCE: Objective structured clinical exam
PICU: Pediatric intensive care unit
