# Supplementary material for: Evaluating a longitudinal point-of-care-ultrasound (POCUS) curriculum for pediatric residents
Source: BMC Med Educ. 2021 Jan 19;21:64. doi: 10.1186/s12909-021-02488-z (PMC7816421; doi:10.1186/s12909-021-02488-z)
Supplement: Supplementary file 1 — Additional file 1. Pediatric Resident Ultrasound Curriculum – Pre-Test. [file 12909_2021_2488_MOESM1_ESM.docx]

**Pediatric Resident Ultrasound Curriculum – Pre-Test**

**1/23/2018**

For data tracking purposes, please fill in the last 5 digits of your cell phone #. _______________

Please fill in your email address _________________________________________________________
(This would be used to communicate future study information with you. All data obtained from this study would be de-identified.)

1. Your colleague shows you several images of an ultrasound exam she just performed. While looking over her images, you notice that she made an adjustment to the machine settings to improve visualization. Which control function was changed from image A to image B?

1. Change from sector to curvilinear probe
2. Increase in total depth
3. Increase in transducer frequency
4. Increase in number of focal zones
5. Increase in total gain
6. Your colleague shows you several images of an ultrasound exam she just performed. While looking over her images, you notice that she made an adjustment to the machine settings to improve visualization. Which control function was changed from image A to image B?

1. Increase depth
2. Increase total gain
3. Use of the zoom function
4. Decrease depth
5. Increase in number of focal zones
6. With respect to the liver parenchyma in this image (asterisk), which number is anechoic?

*****

1. 1
2. 2
3. 3
4. 4
5. The flow represented in this color Doppler image is towards the ultrasound transducer?

1. True
2. False
3. What artifact(s) are present in this image?

1. Shadowing
2. Edge
3. Comet tail
4. Reverberation
5. Edge & shadowing
6. Match the image with the transducer used to obtain that image.

8.

9.

7.

6.

6.______________ Phased array (a)

7.______________ Endocavitary (b)

8.______________ Curvilinear (c)

9.______________ Linear (d)

1. All of the following can be used as a standoff pad except:
   1. Liberal amount of coupling gel
   2. Glove filled with air
   3. Water-bath
   4. Commercially available standoff gel
2. Which of the following accurately describes the ultrasonographic appearance of a tendon?
   1. Hyperechoic with posterior shadow
   2. Diffuse hyperechoic speckles within a Hypoechoic background (transverse view)
   3. Fibrillar pattern of closely spaced, parallel, bright, linear reflections (longitudinal view)
   4. Thin, smooth hypoechoic or anechoic layer
3. Which of the following accurately describes the ultrasonographic appearance of bone?
   1. Thin, smooth hypoechoic or anechoic layer
   2. Fibrillar pattern of closely spaced, parallel, bright, linear reflections (longitudinal view)
   3. Diffuse hyperechoic speckles within a Hypoechoic background (transverse view)
   4. Hyperechoic with posterior shadow
4. Metallic foreign bodies exhibit which characteristic artifact(s) posteriorly?
   1. Comet tail
   2. Grating lobe
   3. Posterior enhancement
   4. Reverberation
   5. a & d
5. Wooden foreign bodies exhibit which characteristic artifact(s) posteriorly?
   1. Comet tail
   2. Grating lobe
   3. Posterior enhancement
   4. Shadowing
   5. b & d
6. When evaluating the skin and superficial soft tissues the optimal transducer is the:
   1. Curvilinear array (3-5 MHz)
   2. Linear array (5-10 MHz)
   3. Phased array (2-5 MHz)
   4. All of the above
7. The classic ultrasound findings for cellulitis include all of the following except:
   1. Increased distance from the skin surface to the underlying fascial planes
   2. Preservation of normal skin architecture
   3. Absence of a discrete fluid-filled collection
   4. Cobblestoning
8. Which of the following is not classically seen when visualizing an abscess with ultrasound?
   1. Irregular, thick-walled appearance
   2. Fluid motion with transducer
   3. Posterior enhancement
   4. Increased central blood flow
9. The easiest location in a long bone to identify a fracture using ultrasound is the:
   1. Metaphysis
   2. Diaphysis
   3. Epiphysis
   4. Articular surface
10. The patient below presents to the ED with a swollen, tender left breast. Which management strategy is appropriate:

- 1. Topical antibiotics
  2. Needle aspiration
  3. Repeat ultrasound in 48 hours
  4. Cold compress

1. The patient below presents to the ED with a painful, red, swollen leg. The picture demonstrates:

- 1. Cobblestoning
  2. Abscess
  3. Deep venous thrombosis
  4. Acoustic enhancement

1. Which letter in the picture represents Morison’s pouch?

A

B

C

D

- 1. A
  2. B
  3. C
  4. D

1. Which letter represents the pleural line?


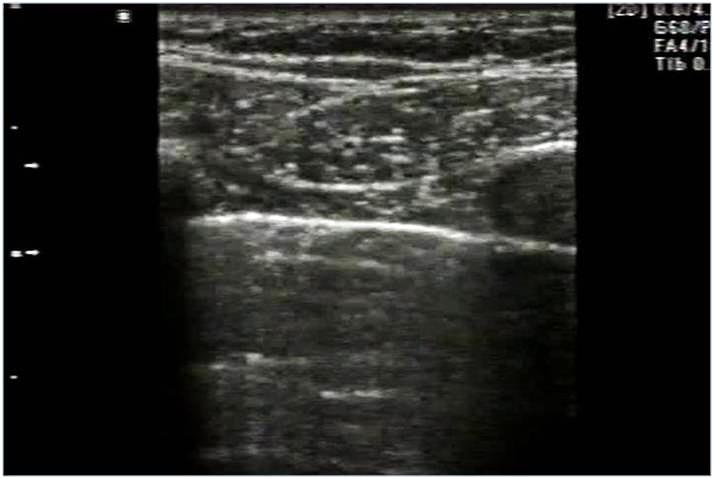


A

B

C

D

- 1. A
  2. B
  3. C
  4. D

1. The picture is an example of a positive FAST?


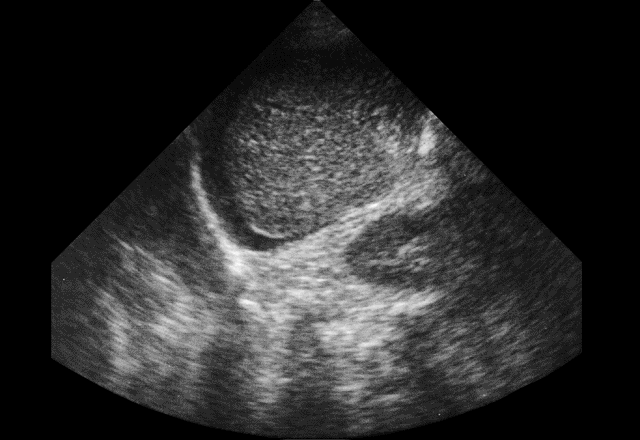


- 1. True
  2. False

1. The FAST exam is most useful for detecting which of the following?
   1. Hemoperitoneum
   2. Renal injury
   3. Intestinal perforation
   4. Diaphragmatic injury
2. Which letter represents the pleural effusion?

A

B

C

D

- 1. A
  2. B
  3. C
  4. D

1. Which of the following views is not part of the basic FAST exam?
   1. Subxyphoid
   2. Perihepatic
   3. Pelvic
   4. Perisplenic
   5. Periumbilical
2. Compared to the right kidney, the left kidney is more:
   1. Anterior and inferior
   2. Posterior and inferior
   3. Anterior and superior
   4. Posterior and superior
3. An obese patient presents after a high speed MVA with a BP 104/66, HR 108. You perform the abdominal views of the FAST exam but are unable to obtain a cardiac view at the subxiphoid level due to patient discomfort and habitus. What would be the next step?
   1. Obtain a chest xray
   2. Continue FAST and skip the cardiac view
   3. Call a trauma surgeon
   4. Attempt a parasternal view
4. Which of the following injuries is most likely to be missed by the FAST exam?
   1. Retroperitoneal hematoma
   2. Mesenteric vein laceration
   3. Grade III splenic laceration
   4. Intraperitoneal bladder rupture
5. This image demonstrates normal lung sliding

- 1. True
  2. False

1. Which of the following maneuvers will not increase the sensitivity of detecting fluid in Morison's pouch?
   1. Serial or repeat FAST exams
   2. Reverse Trendelenberg position
   3. Experienced operator performing the exam
   4. Trendelenberg position

For questions 32 & 33:

A

B

C

D

1. Which letter represents the correct transducer position for a subxiphoid view of the heart?
   1. A
   2. B
   3. C
   4. D
2. Which letter represents the correct transducer position for a parasternal long axis view of the heart?
   1. A
   2. B
   3. C
   4. D
3. In this parasternal long axis view of the heart, global left ventricular contractility is (video on screen)
   1. Normal
   2. Decreased
   3. Increased
4. This echo image shows which common view of the heart?


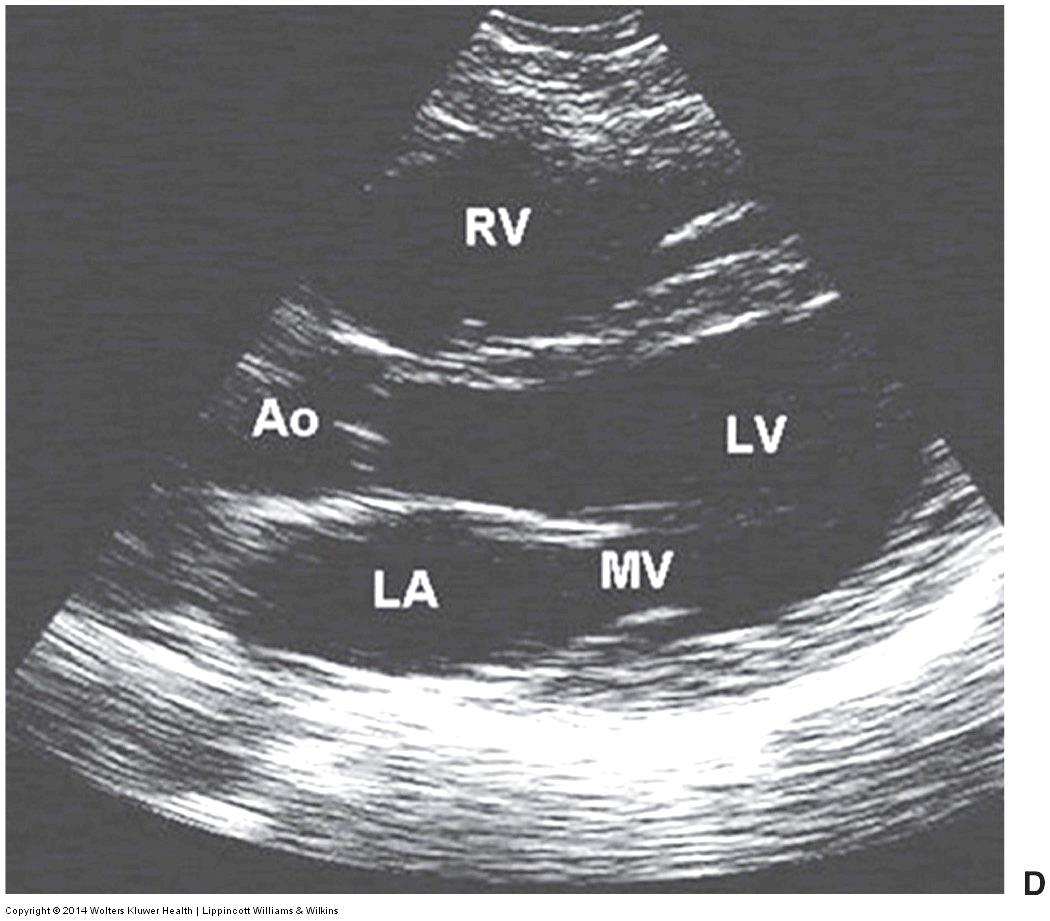


- 1. Parasternal long axis
  2. Parasternal short axis
  3. Apical 4 chamber
  4. Subxiphoid

1. A 17 year old male is found bleeding on the sidewalk. He states that he was attacked with ʻa really big knifeʼ. His pulse is 104/minute and his blood pressure is 86/60 mm Hg. The subxiphoid view of the heart shows what abnormality?


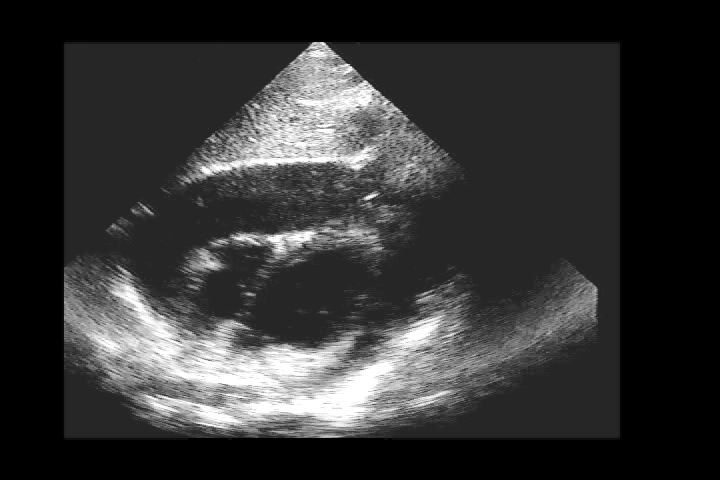


- 1. Peritoneal free fluid
  2. Pleural free fluid
  3. There is no free fluid
  4. Pericardial free fluid

1. The primary goal(s) of bedside emergency echocardiography is (are) immediate evaluation for:
   1. Valvular pathology
   2. Segmental wall motion abnormalities
   3. Pericardial effusions
   4. Gross cardiac contractility
   5. c & d
2. A 19 year old male was noted to collapse on sidewalk. Medics arrived to find the patient in ventricular fibrillation and administered several shocks without return of spontaneous circulation. The patient was intubated, CPR continued and epinephrine and amiodarone administered. On arrival to the Emergency Department after 30 minutes of resuscitative efforts, the patient is in cardiac arrest and his cardiac rhythm is pulseless electrical activity (PEA). The following subxiphoid echocardiogram of the heart is diagnostic of what clinical state? (video on screen)
   1. Refractory ventricular fibrillation
   2. Cardiac standstill
   3. Hypodynamic cardiac contractility with organized movement of mitral and aortic valves
   4. Cardiac tamponade
