# Supplementary material for: Evaluating a longitudinal point-of-care-ultrasound (POCUS) curriculum for pediatric residents
Source: BMC Med Educ. 2021 Jan 19;21:64. doi: 10.1186/s12909-021-02488-z (PMC7816421; doi:10.1186/s12909-021-02488-z)
Supplement: Supplementary file 3 — Additional file 3. Pediatric Resident Ultrasound Curriculum - Pre-course Survey. [file 12909_2021_2488_MOESM3_ESM.docx]

**Pediatric Resident Ultrasound Curriculum - Pre-course Survey**

**1/23/2018**

For data tracking purposes, please fill in the last 5 digits of your cell phone #. _______________

Please fill in your email address _________________________________________________________
(This would be used to communicate future study information with you. All data obtained from this study would be de-identified.)

1. Please circle your year of residency 1 2 3 4

2. What is your age? _________

4. Please circle your gender. Male or Female

5. Have you worked in the Children’s Colorado or Denver Health ED in the last 12 months?

a. Yes

b. No

6. Have you done a residency or internship prior to your pediatric residency?

a. Yes. If so, what type of residency/internship? ____________________

b. No

7. Prior training in point-of-care ultrasound in **GENERAL**. (Please select all that apply)

[ ] None

[ ] Podcasts

[ ] Online course

[ ] Ultrasound course or conference (approximately 1-3 days)

[ ] Rotation or elective (approximately 1 month)

[ ] Bedside teaching and didactics throughout residency (not including current course)

[ ] Bedside teaching and didactics throughout medical school

[ ] Other (please specify) _____________________________________________________

8. Prior training in point-of-care ultrasound on **Soft Tissue** (e.g. cellulitis, abscess, tendon assessment, fractures). (Please select all that apply)

[ ] None

[ ] Podcasts

[ ] Online course

[ ] Ultrasound course or conference (approximately 1-3 days)

[ ] Rotation or elective (approximately 1 month)

[ ] Bedside teaching and didactics throughout residency (not including current course)

[ ] Bedside teaching and didactics throughout medical school

[ ] Other (please specify) _____________________________________________________

8. Prior training in point-of-care ultrasound on **FAST (Focused Assessment with Sonography with Trauma)**. (Please select all that apply)

[ ] None

[ ] Podcasts

[ ] Online course

[ ] Ultrasound course or conference (approximately 1-3 days)

[ ] Rotation or elective (approximately 1 month)

[ ] Bedside teaching and didactics throughout residency (not including current course)

[ ] Bedside teaching and didactics throughout medical school

[ ] Other (please specify) _____________________________________________________

8. Prior training in point-of-care ultrasound on **ECHO**. (Please select all that apply)

[ ] None

[ ] Podcasts

[ ] Online course

[ ] Ultrasound course or conference (approximately 1-3 days)

[ ] Rotation or elective (approximately 1 month)

[ ] Bedside teaching and didactics throughout residency (not including current course)

[ ] Bedside teaching and didactics throughout medical school

[ ] Other (please specify) _____________________________________________________

10. How often do you use point-of-care ultrasound in the clinical setting?

a) Often (>1 time per week)

b) Somewhat often (1-2 times per month)

c) Occasional (<1 time per month)

d) Never

11. How many point-of-care ultrasound exams have you personally performed in medical school and residency?

a) 0-10

b) 11-20

d) 21-30

e) 31-40

f) > 40

12. Comfort level in acquiring and interpreting ultrasound images for **Soft Tissue** exams.

a) Very comfortable

b) Somewhat comfortable

c) Somewhat uncomfortable

d) Very uncomfortable

13. Comfort level in acquiring and interpreting ultrasound images for **EFAST** exams.

a) Very comfortable

b) Somewhat comfortable

c) Somewhat uncomfortable

d) Very uncomfortable

14. Comfort level in acquiring and interpreting ultrasound images for **ECHO** exams.

a) Very comfortable

b) Somewhat comfortable

c) Somewhat uncomfortable

d) Very uncomfortable

15. In the past 3 months, approximately how many **Soft tissue** exams have you done?

1. 0-5
2. 6-10
3. 10-15
4. > 15

16. In the past 3 months, approximately how many **EFAST** exams have you done?

1. 0-5
2. 6-10
3. 10-15
4. > 15

17. In the past 3 months, approximately how many **ECHO** exams have you done?

1. 0-5
2. 6-10
3. 10-15
4. > 15
