# Supplementary material for: Evaluating a longitudinal point-of-care-ultrasound (POCUS) curriculum for pediatric residents
Source: BMC Med Educ. 2021 Jan 19;21:64. doi: 10.1186/s12909-021-02488-z (PMC7816421; doi:10.1186/s12909-021-02488-z)
Supplement: Supplementary file 4 — Additional file 4. Pediatric Resident Ultrasound Curriculum - Post-course Survey. [file 12909_2021_2488_MOESM4_ESM.docx]

**Pediatric Resident Ultrasound Curriculum - Post-course Survey**

**3/6/18**

For data tracking purposes, please fill in the last 5 digits of your cell phone number. _______________

Please fill in your email address _________________________________________________________
(This would be used to communicate future study information with you. All data obtained from this study would be de-identified.)

1. Please circle which ultrasound sessions you attended (circle all that apply):

- 1. Session 1: Knobology & Soft Tissue
  2. Session 2: EFAST & ECHO
  3. Session 3: Resuscitation and conclusion

2. Since starting the ultrasound education (3 months ago), how often do you use point-of-care ultrasound in the clinical setting?

a) Often (>1 time per week)

b) Somewhat often (1-2 times per month)

c) Occasional (<1 time per month)

d) Never

3. Comfort level in acquiring and interpreting ultrasound images for **Soft Tissue** exams.

a) Very comfortable

b) Somewhat comfortable

c) Somewhat uncomfortable

d) Very uncomfortable

4. Comfort level in acquiring and interpreting ultrasound images for **EFAST** exams.

a) Very comfortable

b) Somewhat comfortable

c) Somewhat uncomfortable

d) Very uncomfortable

5. Comfort level in acquiring and interpreting ultrasound images for **ECHO** exams.

a) Very comfortable

b) Somewhat comfortable

c) Somewhat uncomfortable

d) Very uncomfortable

6. In the past 3 months, approximately how many **Soft tissue** exams have you done clinically (on shift or in the clinic)?

1. 0-5
2. 6-10
3. 10-15
4. > 15

7. In the past 3 months, approximately how many **EFAST** exams have you done (on shift or in the clinic)?

1. 0-5
2. 6-10
3. 10-15
4. > 15

8. In the past 3 months, approximately how many **ECHO** exams have you done (on shift or in the clinic)?

1. 0-5
2. 6-10
3. 10-15
4. > 15

9. Did you find the teaching format to be effective?

a) Very effective

b) Somewhat effective

c) Somewhat ineffective

d) Very ineffective

10. Did you find the overall course useful?

1. Yes
2. No

11. What additional type of training do you prefer to be proficient in acquiring and
 interpreting point-of-care ultrasound images? Please select **ALL** that apply.

1. Hands on session
2. Classroom didactic
3. Web-based teaching
4. Review of ultrasound images with expert
5. None (the initial teaching session was sufficient)

12. Did you review the web-based educational resources which were suggested reading for the course?

1. Yes
2. No

13. Please provide any additional feedback below.
