# Supplementary material for: Evaluating a longitudinal point-of-care-ultrasound (POCUS) curriculum for pediatric residents
Source: BMC Med Educ. 2021 Jan 19;21:64. doi: 10.1186/s12909-021-02488-z (PMC7816421; doi:10.1186/s12909-021-02488-z)
Supplement: Supplementary file 5 — Additional file 5. Pediatric Resident Ultrasound Curriculum – OSCE. [file 12909_2021_2488_MOESM5_ESM.docx]

**Pediatric Resident Ultrasound Curriculum – OSCE**

**3/6/2018**

For data tracking purposes, please fill in the last 5 digits of your cell phone #. _______________

Please fill in your email address _________________________________________________________
(This would be used to communicate future study information with you. All data obtained from this study would be de-identified.)

| **Area** | **Criteria** | **Complete** |
| --- | --- | --- |
| RUQ | Correct Transducer ® |  |
|  | Correct Orientation (Z) |  |
|  | Correct Depth (Z) |  |
|  | Correct Gain (Z) |  |
|  | Morrison’s Pouch |  |
|  | Liver Tip |  |
|  | Inf Pole of Kidney |  |
|  | Identifies lack of FF |  |
|  |  |  |
| LUQ | Correct Transducer ® |  |
|  | Correct Orientation (Z) |  |
|  | Correct Depth (Z) |  |
|  | Correct Gain (Z) |  |
|  | Splenorenal Recess |  |
|  | Spleen tip |  |
|  | Diaphragm |  |
|  | Inf pole L kidney |  |
|  | Identifies lack of FF |  |
|  |  |  |
| Pericardium | Correct Transducer ® |  |
|  | Correct Orientation (Z) |  |
|  | Correct Depth (Z) |  |
|  | Correct Gain (Z) |  |
|  | Subxyphoid View anterior pericardium |  |
|  | Subxypoind View Posterior Pericardium |  |
|  | Identifies Lack of FF |  |
|  |  |  |
| Bladder | Correct Transducer ® |  |
|  | Correct Orientation (Z) |  |
|  | Correct Depth (Z) |  |
|  | Correct Gain (Z) |  |
|  | Visualizes entire bladder Longitudinal |  |
|  | Visualizes bladder transverse |  |
|  | Identifies lack of FF |  |
|  |  |  |
| TOTAL |  |  |
